# The Lived Experience of Young Adult Cancer Survivors after Treatment: A Qualitative Study

**DOI:** 10.3390/nu15143145

**Published:** 2023-07-14

**Authors:** Sylvia L. Crowder, Rachel Sauls, Lisa M. Gudenkauf, Christy James, Amber Skinner, Damon R. Reed, Marilyn Stern

**Affiliations:** 1 Department of Health Outcomes and Behavior, H. Lee Moffitt Cancer Center, Tampa, FL 33612, USA; 2 College of Public Health, University of South Florida, Tampa, FL 33620, USA; 3 Non-Therapeutic Research Operations, H. Lee Moffitt Cancer Center, Tampa, FL 33612, USA; 4 Adolescent and Young Adult Program, H. Lee Moffitt Cancer Center, Tampa, FL 33612, USA; 5 Department of Individualized Cancer Management, H. Lee Moffitt Cancer Center, Tampa, FL 33612, USA; 6 College of Behavioral and Community Sciences, University of South Florida, Tampa, FL 33620, USA

**Keywords:** young adult, cancer, survivorship, diet, nutrition, exercise, psychosocial functioning, qualitative research

## Abstract

Objective: The purpose of this qualitative study was to compare the lived experiences among extended (one year or less post-treatment) and long-term (three years or more post-treatment) young adult (YA) cancer survivors (ages 18–39 years old). Methods: Two trained researchers conducted semi-structured interviews inquiring about the overall lived experience of *N* = 24 YA cancer survivors (*n* = 12 extended and *n* = 12 long-term). The same two researchers independently completed line-by-line coding and thematic content analysis. Results: Interviews lasted an average of 41 min and revealed common themes of *symptoms*, *psychosocial concerns*, *coping*, *and changes in health behaviors* (e.g., *nutrition and physical activity*). All participants discussed symptoms impairing their quality of life and affecting their fear of recurrence. Specific psychosocial concerns among extended survivors were appearance-related (e.g., hair loss, weight gain) whereas concerns among long-term survivors included job loss, fertility, and financial stress. Coping strategies described by extended survivors were often distraction-based (e.g., watching television to “escape”), while long-term survivors described more active coping strategies (e.g., yoga, meditation, and seeking support from family and friends). Most survivors reflected on limited physical activity or unhealthy eating during treatment; however, nearly all declared healthy eating and physical activity post-treatment to improve well-being. Conclusions: YA cancer survivors report differing symptoms, psychosocial concerns, and coping strategies across time since treatment. While survivors reported challenges with physical activity and nutrition during treatment, nearly all emphasized the importance of these health behaviors post-treatment. Thus, health behavior interventions could represent a preferred approach to address post-treatment challenges and improve quality of life for YA survivors.

## 1. Background

Survival rates among young adults (YA) ages 18–39 years old now exceed 85% for all cancer types [1,2]. As this survivorship cohort continues to grow, so do the needs for YA post-treatment care [3,4,5]. Compared to peers without cancer, YA survivors experience unique challenges related to reaching developmental milestones (e.g., completing educational goals and career advancement), adapting to post-treatment life [6], and long-term health management, especially given the higher prevalence of chronic disease, disability, and reduced quality of life [7]. Compared to older adults with cancer, YAs experience unique developmental challenges of young adulthood while simultaneously navigating cancer treatment [8,9,10,11], and YAs report worse quality of life than cancer survivors in other age cohorts [12,13]. Indeed, in a recent systematic review, treatment effects were more prominent among YA cancer survivors compared to pediatric cancer survivors [14], highlighting the importance of addressing the specific unmet needs of YA survivors. Yet, the specific psychosocial, behavioral, and supportive care concerns and needs of YA survivors remain underexplored.

Most research on the unmet needs and lived experience of YA cancer survivors has primarily focused on survivors of childhood cancer [15,16,17,18]. However, the physical and psychological effects of a cancer diagnosis during young adulthood differ from the effects of a childhood diagnosis [19]. For instance, previous research has suggested that quality of life may return to a “new normal” after cancer treatment more easily among children than young adults [20,21]. Furthermore, YA program development has been predominantly informed by pediatric centers and practitioners, yet nearly all YA survivors receive treatment at adult cancer centers [22]. Adult cancer centers have limited expertise or infrastructure to address the unique care needs of the YA patient population [22]. Thus, research on the lived experience of YA survivors is critically needed to identify specific, unmet needs for YA across the survivorship trajectory and to inform supportive care interventions post-treatment, when unmet needs are at their highest [23]. Specifically, understanding the overlapping and distinct needs of YA survivors (e.g., quality of life, behavioral (nutrition and physical activity), and psychosocial concerns in extended survivorship (end of initial cancer treatment and months thereafter) [24] and long-term survivorship (years have passed since cancer treatment ended) [24] may empower adult cancer centers, practitioners, and researchers to optimize post-treatment care for YA survivors [25].

Thus, this study aimed to characterize the lived experience of YA survivors and identify unmet needs across the survivorship trajectory, comparing YAs in the extended survivorship versus long-term survivorship periods. The identification of priority issues in the extended and long-term survivorship periods will inform the development of future, need-specific interventions with the overall goal of improving the quality of life of YA cancer survivors.

## 2. Methods

### 2.1. Ethical Approval

This study was performed in line with the principles of the Declaration of Helsinki and was approved by the Advarra Institutional Review Board of Moffitt Cancer Center (Pro00053494). All participants were informed of the purpose and procedures of the study and were informed that study participation was voluntary, with participants retaining the right to withdraw at any time without explanation. All participants provided verbal informed consent prior to data collection. Patient health information was anonymized and assigned a number code to ensure confidentiality. No individuals are identifiable in any presentation of results.

### 2.2. Research Design and Participants

This was a semi-structured qualitative interview study with English-speaking YA cancer survivors of any cancer type and any gender, race, or ethnicity treated at an NCI-designated comprehensive center. Inclusion criteria included: (1) diagnosed between 18 and 39 years old; (2) less than one-year post-treatment OR three years or more post-treatment; and (3) able to read and speak English. Survivorship periods are typically explained in year-long increments (i.e., 1 year, 3 years post-treatment) [26], in part due to differences in follow-up and surveillance approaches over time post-treatment. We further limited recruitment to participants aged ≤ 40 years old to ensure that participants were as close to the AYA survivorship period as possible. Participants were recruited via virtual flyers emailed to patients on the cancer institute’s Young Adult ListServ, and recruitment concluded when data saturation was reached. Our recruitment goal was to obtain data saturation, when no new themes occur, in each group (i.e., extended vs. long-term survivors) [27,28,29,30].

### 2.3. Data Collection

Participants completed a brief questionnaire of age at diagnosis, weight at diagnosis, current weight, height, gender, race/ethnicity, education level, cancer type, cancer stage, treatment, comorbidities, and time since treatment completion.

Semi-structured Zoom^®^ interviews were audio recorded and conducted by the first and second authors from August 2021 to February 2022. Interviews were chosen over focus groups as we were interested in learning about individual preferences and experiences rather than group consensus. A qualitative methodology was chosen to better understand the lived experience and needs of young adult cancer survivors rather than a quantitative methodology for specific hypothesis testing. Interviewers were trained to provide only minimal verbal input and prompt only when appropriate [31]. Audio recordings were professionally transcribed verbatim by GMR Transcription Services, Inc., and checked for accuracy to ensure that transcripts represented a complete account of participant responses.

An interview guide was utilized and developed to capture themes represented in literature reviews in combination with input from a team of young adult cancer experts and researchers with clinical experience in the field (example questions provided in Supplementary Table S1). Probes within the interview permitted participants to raise unanticipated issues and allowed interviewers flexibility to follow such leads. Interview transcripts and audio files were stored on a password-protected drive. A USD 25 e-gift card was offered to all participants who completed the semi-structured qualitative interview.

### 2.4. Data Analysis

Dedoose™ Version 9.0 [32], a qualitative software web application, was used for qualitative coding according to the six-step thematic analysis approach as outlined by Braun and Clarke [33]. The grounded theory method was used as an inductive process whereby theoretical insights are generated from data, in contrast to deductive research where theoretical hypotheses are tested via data collection [33]. This was chosen to provide a rich description of the data and identify themes at an explicit level using a realistic approach [33]. The first and second authors who conducted all Zoom^®^ interviews re-familiarized themselves with the data by reading all verbatim transcripts. The two authors then conducted line-by-line coding with statements thought to be related to symptoms, psychosocial concerns, coping strategies, and changes in health behaviors (e.g., nutrition and physical activity). The codes were refined and amended until a single list was agreed upon in formal discussion. Themes were further reviewed, refined, named, and given a written description.

## 3. Results

**Figure 1:**
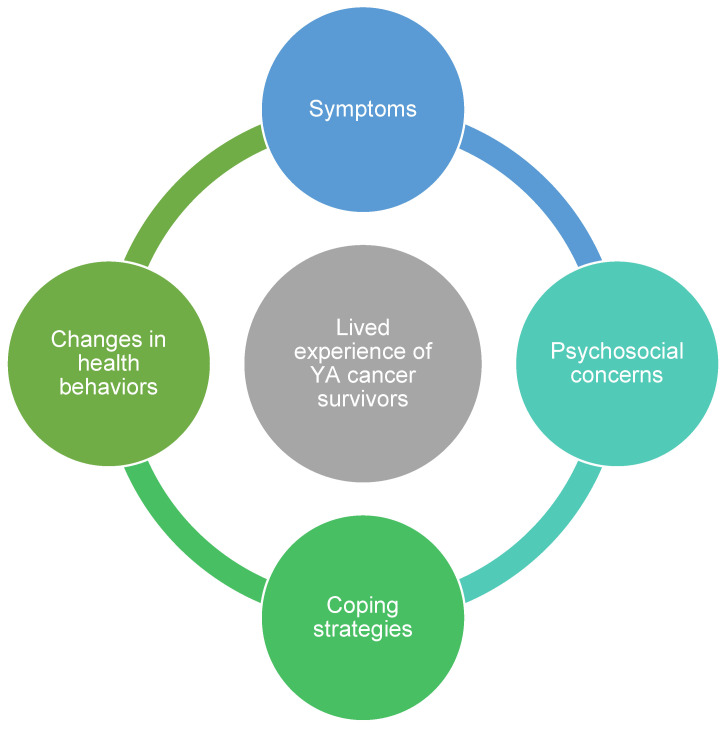
Qualitative themes of semi-structured interviews about the lived experience of young adult (YA) cancer survivors *(N* = 24) after treatment completion.

Among the 24 total YA participants interviewed (*n* = 12 extended survivors and *n* = 12 long-term survivors), the majority were non-Hispanic (*n* = 21, 88%) white (*n* = 13, 54%) females (*n* = 20, 83%). Additional participant demographics are shown in Table 1. Interviews lasted on average 41 min (range 20–68 min). Common themes included treatment-related *symptoms, psychosocial concerns, coping strategies, and changes in health behaviors (e.g., nutrition and physical activity)* from before cancer to current day habits, as seen in Figure 1. Similarities and differences among extended survivors and long-term survivors across these four common themes are described below and presented in Table 2.

## 4. Symptoms

Among the 24 participants, 22 YAs (*n* = 11 extended survivors and *n* = 11 long-term survivors) reported experiencing symptoms (e.g., long-term or late effects) from treatment, such as fatigue (*n* = 12), resulting in quality of life impairments. For extended survivors, symptoms included hair loss (*n* = 3), fatigue (*n* = 7), and mobility limitations (e.g., pain with walking or instability; *n* = 4). Long-term survivors discussed concerns related to life stress (e.g., fear of the unknown/feeling left behind; *n* = 5) and hormonal changes (e.g., menopausal symptoms, weight gain; *n* = 3).

“Yeah. I would say fatigue... I think I have too much thyroid in my body still. So, I think that’s contributing to things. For my physical appearance, yeah, I’d say my body has changed, and of course I have a scar now.”

—1025, female, extended survivor.

“I felt limited physically and hopeless. And sometimes even now today at 26, even though I’m okay, now I have my days when I kinda feel like I’m falling behind because my energy is not like what it used to be, and people will never understand that.”

—1015, female, long-term survivor.

### 4.1. Nutrition

Extended survivors (*n* = 10) and long-term survivors (*n* = 11) reported impaired nutritional intake both during and after treatment, including loss of taste (*n* = 4), nausea (*n* = 8), diarrhea (*n* = 4), pain with eating (*n* = 6), and dietary restrictions (e.g., mouth sores; *n* = 6). Long-term survivors also reported taste alterations (e.g., metallic taste, change in food preference) as a nutritional symptom (*n* = 3). Extended survivors (*n* = 3) and long-term survivors (*n* = 3) reported nutritional impairments resolving after treatment completion.

“Yeah, during and after treatment [4 months], I just didn’t have an appetite for anything, and it was really bad when I was in the hospital and had all those medications to take. It was just a lot of stress on my body, and I just hated the idea of eating.”

—1022, female, extended survivor.

“Everything just kinda tasted a little funky. It was more like a dampened flavor like, things don’t have as much flavor as I expect them to. And right now, I would say some things that are really creamy come off really waxy, so it’s a little bit of a different texture type thing, too. Just makes eating harder.”

—1021, male, extended survivor.

### 4.2. Physical Activity

Symptoms impacting physical activity were common in both the extended survivor (*n* = 8) and long-term survivor (*n* = 6) groups. Physical activity challenges included reduced muscle mass (*n* = 4), impaired daily physical functioning (e.g., crossing legs or bone pain; *n* = 6), and fatigue (e.g., reduced endurance or being out of breath; *n* = 12). Several participants (*n* = 7) reported struggling to “get back” to the level of physical activity they had before cancer treatment.

“I couldn’t do anything physical because I was at such a high level of pain. And I was exhausted all the time, just so tired it was hard to even think about being physically active.”

—1012, female, extended survivor.

“More of the mobility issue than the appearance but, I mean, both kinda bothers me a little bit, just not being able to bend my leg and build that muscle back up. My leg is very atrophied, so it’s a little bit smaller than my other leg and sometimes that bothers me, but I’m just grateful to have my leg.”

—1019, female, long-term survivor.

## 5. Psychosocial Concerns

All participants reported psychosocial concerns (*n* = 24), such as anxiety (e.g., cancer recurrence, treatment anxiety; *n* = 20), worries about appearance (*n* = 24), fertility concerns (*n* = 5), reduced self-esteem (*n* = 22), and feeling self-conscious (*n* = 6). Every participant mentioned an appearance-related concern, specifically skin scarring (*n* = 11), hair loss (*n* = 18), and body image concerns (*n* = 8). Extended survivors (*n* = 9) reported actively struggling with appearance concerns (*n* = 9), whereas long-term survivors reported appearance-related concerns resolving over time (*n* = 7). Weight-related concerns were reported among both extended survivors (*n* = 12) and long-term survivors (*n* = 9). Both groups reported concerns such as fatigue (*n* = 12) and feeling self-conscious (*n* = 6).

### 5.1. Anxiety

“And now, I’m also thinking that maybe the surgery wasn’t as successful as we initially thought, or is it because I had a weakened dose of chemo? So, now I’m in this guessing game, and I also have so many specialists’ appointments coming up to clear up other things that are outstanding, that it’s just a little overwhelming.”

—1017, male, extended survivor.

“Just a huge fear of re-occurrence. I will say I am so much more aware of anything I feel. Any symptom that I have could be related to cancer and so I always err on the side of caution, it is just a constant level of stress and anxiety. When I had a period come back, which it has been years, I thought cervical cancer… good or bad, I end up putting myself through more than the average person whenever I have any symptom.”

—1002, female, long-term survivor.

### 5.2. Appearance

“Overall, physical appearance [is my biggest concern], I think. Because it affects everybody differently, but it’s more … mental. You lose your hair. You lose all the features that made you, you, and now you’re somebody else. My jawline. I used to have a jawline, things like that. But now, it’s just a round, pudgy face and it really just wasn’t what I wanted.”

—1023, male, extended survivor.

“The biggest setback for me was obviously my hair appearance-wise. Losing my hair was a little challenging… It is much thinner than prior chemo, which I struggle with a little bit. But I have hair. So, that’s amazing.”

—1016, female, long-term survivor.

### 5.3. Fertility

“So, one of the biggest things, and I’m sure you’re aware of this, but chemotherapy can negatively impact your fertility. I haven’t had kids yet. So, that’s a really big concern for me. The doctors tell me that some men do get their fertility back at some point. So, that’s one of my concerns.”

—1004, male, long-term survivor.

“I mean, a lot of people don’t tell you. Everyone thinks, well, you’re not gonna be able to have kids, but they don’t tell you you’re gonna be in menopause which affects your fertility—I think you don’t really think about it. You just kind of say, “Oh. Other people don’t have kids.” But, no. You’re in full ovarian failure. You’re going through menopause.”

—1013, female, long-term survivor.

### 5.4. Additional Concerns

Additional, less commonly reported concerns included job-related stresses (*n* = 5), such as fear of being fired, health insurance coverage, and related financial concerns, primarily in the long-term survivor group (*n* = 4). Extended survivors also reported specific concerns, such as medication side effects (*n* = 5) and isolation related to quarantining for the COVID-19 pandemic (*n* = 4).

“I didn’t want to get out of my house, did not get out of my bed, and, also, after or close to when I was recovering, it was hard for me to find a job. So, financially after, when you’re a young adult with cancer, when you had cancer that happens like you have a hard time finding work again because my current job, when I had that at the time let me go, and I had a hard time for the past four years. And I’m okay now, but it was really difficult.”

—1015, female, long-term survivor.

## 6. Coping Strategies

Almost all participants (*n* = 23) described coping strategies including positive and negative strategies, such as seeking social support (*n* = 16), physical activity (*n* = 7), or eating to cope with feelings (*n* = 4). Types of social support included family (*n* = 12), friends (*n* = 10), and social media support groups (*n* = 8). More long-term survivors (*n* = 4) than extended survivors (*n* = 3) reported coping through physical activity (e.g., walking, going to the gym, and dancing). More extended survivors (*n* = 6) reported coping through eating and mentioned overeating, eating out, or cooking at home as a distraction. Additional coping strategies of YA survivors included mindfulness (yoga, meditation, journaling; *n* = 8), using distractions (watching Netflix, listening to music; *n* = 4), and humor (*n* = 2).

### Social Support

“I have a really good network of friends and family that kept me together. Whenever there was issues, I would focus on other things instead of myself or I would always think there’s somebody out there who’s worse, somebody that doesn’t have what I have. So, I was able to put that in my mind and it always helped.”

—1023, male, extended survivor.

“Honestly, I went to Facebook… and I looked for support groups because I would tell my oncologist, and I just felt like they didn’t get it. They would just look at me like I’ve got four eyes, and I’m like, ‘No, this is really concerning to me.’ So, I just looked to Facebook and support groups who understood where I was coming from, and it dramatically helped because I was like, ‘I’m not crazy! You understand me! I’m not crazy!’ So, ‘til this day, I still use them. I’ve met a lot of cool friends there.”

—1007, female, long-term survivor.

## 7. Changes in Nutrition and Physical Activity

Participants were asked to reflect upon changes in health behaviors throughout their cancer survivorship journey. When reflecting upon the period prior to cancer treatment, *n* = 12 participants (*n* = 7 extended survivors and *n* = 5 long-term survivors) reported previously healthy eating and *n* = 18 reported previously being physically active (*n* = 9 extended survivors and *n* = 9 long-term survivors). Reflecting upon the treatment period, only *n* = 2 participants reported healthy eating and *n* = 3 reported physical activity. The number of participants engaged in healthy behaviors increased after treatment. With regard to the post-treatment period, *n* = 22 participants reported healthy eating (*n* = 11 extended survivors and *n* = 11 long-term survivors), and *n* = 16 reported participating in scheduled physical activity. More long-term survivors (*n* = 9) than extended survivors (*n* = 7) reported being physically active. All twelve participants who reported unhealthy eating before treatment reported consuming a “healthier” diet after treatment completion. The two participants who reported unhealthy eating post-treatment stated that they were unable to eat healthy foods due to permanent taste alterations and due to eating fast food while traveling far distances for medical appointments.

“I was a college athlete, so I love weightlifting. I just don’t have that strength fully back yet to be able to do it. So, the most I’ll do is some lower-weight dumbbells or something like that but definitely used to have longer more high-level intensity workouts whereas now it’s more just like a maintenance getting my body moving type of thing. I work out probably like 45 min to an hour a day.”

—1010, female, extended survivor.

“I wasn’t really physically active before. I used to walk around campus or things like that, but I never really did anything. I used to go to the gym every now and then. I feel like I’m more active now just because I’m putting in the effort. I do Pilates. I do rowing. I walk my dog 30 min a day, things like that. So, I feel like I’m more active now.”

—1003, female, long-term survivor.

## 8. Discussion

The four qualitative themes—symptoms, psychosocial concerns, coping strategies, and health behavior changes (e.g., changes in nutrition and physical activity)—capture the lived experience of YA cancer survivors. This study adds to the existing literature by specifically investigating similarities and differences in the lived experience of YA survivors in the year after treatment (extended survivors) compared to YA survivors three years or more post-treatment (long-term survivors). Extended survivors reported more impairments to their overall quality of life (e.g., appearance, fatigue, medication side-effects) and more acute concerns (e.g., body image, weight changes, hair loss). In comparison, long-term survivors (*n* = 11) commonly reported late effects of treatment (e.g., mucositis, post-traumatic stress, and mobility limitations) and more chronic concerns (e.g., ability to find and hold a job, fertility concerns, lasting financial burden) as part of their lived experience. Long-term survivors also reported developing more active coping strategies (e.g., seeking social support through family/friends, physical activity) to adapt to post-treatment life. Interestingly, while YA survivors reported declines in nutrition and physical activity during treatment, nearly all emphasized the importance of nutrition and physical activity post-treatment, regardless of the time since treatment.

The literature is mixed as to whether YA survivors’ health behaviors are worse or better than their healthy peers [34,35]. This study helps elucidate fluctuations in health behaviors across time, which may be contributing to mixed results in the literature. Moreover, given YA interest in nutrition and physical activity regardless of time since treatment completion, supportive care services could be tailored to provide health behavior interventions during or immediately following treatment to meet survivorship needs by promoting the adaptation and maintenance of healthy behavior coping strategies. Potential benefits of health behavior interventions are supported by results of prior studies demonstrating the efficacy of health behavior intervention programs at different stages in the cancer journey ranging from active therapy to survivorship [36,37]. Furthermore, given the most reported coping strategies for seeking social support, including spending quality time with family (*n* = 12) and friends (*n* = 10) and engaging in social media support groups (*n* = 8), future research should investigate whether remote health behavior interventions utilizing apps or social media platforms with a caregiver component (e.g., family/friends) would be of interest for this population. Remote health behavior interventions may be particularly preferable for YA survivors given their responsibilities and stresses (e.g., raising young children, starting a career/seeking work, living with late effects).

Previous studies have focused on challenges immediately following a cancer diagnosis [38,39,40], while this study offers a focused examination of YA experiences post-treatment and over time after treatment completion. Although study findings are consistent with studies of childhood cancer survivors in many domains, results varied significantly. For instance, YAs in our study reported seeking social support from family and friends as the most frequent coping strategy. This result is similar to a study of Latino childhood cancer survivors who reported family support long after treatment [16]. In contrast, Swedish families in one study reported difficulty maintaining family support for the childhood cancer survivor upon treatment completion, leaving some of these children feeling “forgotten” [41].

### Study Limitations

The current study has notable strengths and limitations. Prior studies predominately focused on the lived experience of childhood cancer survivors, while our study contributed to the literature by focusing on the unique concerns of YA survivors. Our findings are based on young adult YA cancer survivors’ own voices as they articulate their lived experiences and needs. Interviews were conducted via Zoom^®^, providing scheduling flexibility; patients did not need to make special arrangements to come to an in-person interview at the NCI-designated comprehensive cancer center. To overcome the potential barrier of needing digital access to participate in the study, a phone interview was offered if a Zoom^®^ interview would not have been feasible; no participants requested that option. Participants varied by cancer type; thus, the generalizability of findings may not be specific to one cancer population. As a small convenience sample was collected in an urban geographical region during the COVID-19 pandemic, the generalizability may be limited. Given the specific stressors of the COVID-19 pandemic, isolation concerns (*n* = 4) may also have been particularly heightened. We were unable to blind participants to the research question; thus, it is possible individuals who chose to participate represented those YA survivors with the highest unmet needs. Finally, this study only included English-speaking YA cancer survivors. Future studies should expand to include other languages, capturing lived experiences of YAs across various cultural groups and promoting the design of culturally relevant and tailored interventions.

## 9. Conclusions

The risks, complex needs, and enduring challenges of survivorship among YA cancer survivors highlight the importance of better understanding the lived experience and unmet needs of YA survivors in the extended and long-term survivorship phases. The results of the current study showed that YA cancer survivors report varying symptoms, psychosocial concerns, and coping strategies across time after treatment completion. Interestingly, while survivors reported declines in nutrition and physical activity during treatment, nearly all emphasized the importance of these health behaviors post-treatment, regardless of the time since treatment completion. Thus, nutrition and physical activity interventions may represent a preferred approach to address physical symptoms and psychosocial concerns and improve YA quality of life. Overall, our qualitative results provide helpful direction for developing interventions that could be integrated into clinical practice to improve the lived experience of YA cancer survivors after treatment.

## Figures and Tables

**Table 1:** Characteristics of young adult cancer survivors (*N* = 24).

	Extended Survivors (*n* = 12)	Long-term Survivors (*n* = 12)	All Participants (*N* = 24)
**Age:** Mean ± SD (range), years			
At diagnosis	31.2 ± 6.5 (20–39)	25.6 ± 4.9 (19–32)	27.8 ± 6.3 (19–39)
At study enrollment	31.4 ± 6.1 (21–40)	32.1 ± 5.3 (26–40)	31.8 ± 5.6 (21–40)
**Body Mass Index:** Mean ± SD [range], kg/m^2^			
At diagnosis	28.2 ± 6.2 (22–43)	25.7 ± 6.7 (19–41)	27.0 ± 8.3 (19–43)
At study enrollment	29.7 ± 6.5 (21–43)	28.8 ± 8.0 (20–44)	29.2 ± 9.1 (20–44)
**Female Gender:** *n* (%)	9 (75)	11 (92)	20 (83)
**Non-Hispanic Ethnicity:** *n* (%)	10 (83)	11 (92)	21 (88)
**White Race:** *n* (%)	7 (58)	6 (50)	13 (54)
**Education:** *n* (%)			
Some college	0 (0)	5 (42)	5 (21)
College degree	8 (67)	4 (33)	12 (50)
Some graduate school	1 (8)	1 (8)	2 (8)
Graduate school degree	3 (25)	2 (17)	5 (21)
**Cancer Diagnosis:** *n* (%)			
Acute lymphoblastic leukemia	1 (8)	0 (0)	1 (4)
Acute myeloid leukemia	1 (8)	2 (17)	3 (12)
Bladder	1 (8)	0 (0)	1 (4)
Breast	2 (17)	2 (17)	4 (16)
Colorectal	1 (8)	0 (0)	1 (4)
Hodgkin lymphoma	2 (17)	2 (17)	4 (16)
Melanoma	0 (0)	1 (8)	1 (4)
Non-Hodgkin lymphoma	1 (8)	1 (8)	2 (8)
Ovarian	1 (8)	0 (0)	1 (4)
Sarcoma	0 (0)	2 (17)	2 (8)
Testicular	0 (0)	1 (8)	1 (4)
Thyroid	2 (16)	1 (8)	3 (12)
**Cancer stage:** *n* (%)			
Stage 0	2 (17)	1 (8)	3 (13)
Stage I	2 (17)	1 (8)	3 (13)
Stage II	1 (8)	2 (17)	3 (13)
Stage III	2 (17)	1 (8)	3 (13)
Stage IV	2 (17)	2 (17)	4 (16)
No stage; deemed aggressive	2 (17)	2 (17)	4 (16)
Prefer not to answer	1 (8)	3 (25)	4 (16)
**Therapy received:** *n* (%)			
Chemotherapy	4 (34)	2 (17)	6 (25)
Surgery	2 (17)	2 (17)	4 (16)
Chemotherapy + Radiation	1 (8)	1 (8)	2 (8)
Chemotherapy + Surgery	1 (8)	1 (8)	2 (8)
Radiation + Surgery	1 (8)	0 (0)	1 (4)
Chemotherapy + Immunotherapy	1 (8)	0 (0)	1 (4)
Chemotherapy + Immunotherapy + Surgery	0 (0)	1 (8)	1 (4)
Chemotherapy + Hormonal + Surgery	0 (0)	1 (8)	1 (4)
Chemotherapy + Radiation + Surgery	0 (0)	1 (8)	1 (4)
Chemotherapy + Radiation + Immunotherapy + Surgery	1 (8)	2 (17)	3 (13)
Prefer not to answer	1 (8)	1 (8)	2 (8)
**Comorbidities:** *n* (%)			
None	9 (75)	8 (67)	17 (71)
Obesity	2 (17)	3 (25)	5 (21)
Hypertension	1 (8)	0 (0)	1 (4)
Obesity + High Cholesterol	0 (0)	1 (8)	1 (4)

Note. Percentages may not sum to 100 due to rounding.

**Table 2:** Similarities and differences among the lived experiences of extended (*n* = 12) and long-term (*n* = 12) young adult cancer survivors.

Themes	Extended Survivors	Both Groups	Long-Term Survivors
**Symptoms**	Pain, immobility, medication side effects (e.g., hair loss)	Fatigue, dietary restrictions (e.g., taste alterations, sensitivity to certain foods)	Fear of unknown, life stress (e.g., maintaining long-term relationships), hormonal changes (e.g., early onset menopause)
**Psychosocial** **concerns**	Acute concerns (e.g., hair loss, scarring), body image (e.g., appearance, weight fluctuations), COVID-19 isolation, medication side-effects	Treatment anxiety, fear of recurrence, feeling self-conscious, impaired quality of life	Chronic concerns (e.g., ability to find and keep a job, fertility concerns, financial burden)
**Coping strategies**	Distraction-based “negative” coping strategies (e.g., snacking, listening to music)	Mindfulness, humor, engaging in social media support groups	Action-based coping “positive” strategies (e.g., seeking social support, physical activity)
**Changes in health behaviors**	Immobility concerns limiting some physical activity and prioritizing healthy eating as a result	Value for the importance of nutrition and physical activity post-treatment	Engaging in physical activity and healthy eating behaviors

## Data Availability

The data presented in this study are available on request from the corresponding author. The data are not publicly available in order to maintain participant confidentiality due to the sensitive information resulting from the qualitative interviews.

## Supplementary Materials

The following supporting information can be downloaded at: https://www.mdpi.com/article/10.3390/nu15143145/s1, Supplemental Table S1. Interview Question Examples.
