# Supplementary material for: The Lived Experience of Young Adult Cancer Survivors after Treatment: A Qualitative Study
Source: Nutrients. 2023 Jul 14;15(14):3145. doi: 10.3390/nu15143145 (PMC10385438; doi:10.3390/nu15143145)
Supplement: Supplementary file 1 [file nutrients-15-03145-s001.zip › nutrients-2445895-supplementary.pdf]

Table S1. Interview Question Examples.

Question
What symptoms, either related to the cancer or side-effects of treatment, did you experience?<br>Did you have any symptoms or side-effects that impacted your ability to eat a balanced diet? If so, which ones?<br>o PROBE: How did they impact you?<br>o PROBE: How often did you experience them?
Did you have any symptoms or side-effects that impacted your ability to participate in physical activity? If so, which ones?<br>o PROBE: How did they impact you?<br>o PROBE: How often did you experience them?
What parts of your life were most impacted by your cancer experience?<br>o PROBE: What parts of your life were most impacted by cancer treatment?
